# Serum Fatty Acids, Desaturase Activities and Abdominal Obesity – A Population-Based Study of 60-Year Old Men and Women

**DOI:** 10.1371/journal.pone.0170684

**Published:** 2017-01-26

**Authors:** Zayed D. Alsharari, Ulf Risérus, Karin Leander, Per Sjögren, Axel C. Carlsson, Max Vikström, Federica Laguzzi, Bruna Gigante, Tommy Cederholm, Ulf De Faire, Mai-Lis Hellénius, Matti Marklund

**Affiliations:** 1 Department of Public Health and Caring Sciences, Clinical Nutrition and Metabolism, Uppsala University, Uppsala, Sweden; 2 Unit of Cardiovascular Epidemiology, Institute of Environmental Medicine, Karolinska Institutet, Stockholm, Sweden; 3 Division of Family medicine, Department of Neurobiology, Care Sciences and Society, Karolinska Institutet, Stockholm, Sweden; 4 Division of Cardiovascular Medicine, Department of Clinical Sciences, Danderyds Hospital, Karolinska Institutet, Stockholm, Sweden; 5 Cardiology Unit, Department of Medicine, Karolinska Institutet, Karolinska University Hospital, Stockholm, Sweden; Universita degli Studi di Milano, ITALY

## Abstract

Abdominal obesity is a key contributor of metabolic disease. Recent trials suggest that dietary fat quality affects abdominal fat content, where palmitic acid and linoleic acid influence abdominal obesity differently, while effects of n-3 polyunsaturated fatty acids are less studied. Also, fatty acid desaturation may be altered in abdominal obesity. We aimed to investigate cross-sectional associations of serum fatty acids and desaturases with abdominal obesity prevalence in a population-based cohort study. Serum cholesteryl ester fatty acids composition was measured by gas chromatography in 60-year old men (n = 1883) and women (n = 2015). Cross-sectional associations of fatty acids with abdominal obesity prevalence and anthropometric measures (e.g., sagittal abdominal diameter) were evaluated in multivariable-adjusted logistic and linear regression models, respectively. Similar models were employed to investigate relations between desaturase activities (estimated by fatty acid ratios) and abdominal obesity. In logistic regression analyses, palmitic acid, stearoyl-CoA-desaturase and Δ6-desaturase indices were associated with abdominal obesity; multivariable-adjusted odds ratios (95% confidence intervals) for highest versus lowest quartiles were 1.45 (1.19–1.76), 4.06 (3.27–5.05), and 3.07 (2.51–3.75), respectively. Linoleic acid, α-linolenic acid, docohexaenoic acid, and Δ5-desaturase were inversely associated with abdominal obesity; multivariable-adjusted odds ratios (95% confidence intervals): 0.39 (0.32–0.48), 0.74 (0.61–0.89), 0.76 (0.62–0.93), and 0.40 (0.33–0.49), respectively. Eicosapentaenoic acid was not associated with abdominal obesity. Similar results were obtained from linear regression models evaluating associations with different anthropometric measures. Sex-specific and linear associations were mainly observed for n3-polyunsaturated fatty acids, while associations of the other exposures were generally non-linear and similar across sexes. In accordance with findings from short-term trials, abdominal obesity was more common among individuals with relatively high proportions of palmitic acid, whilst the contrary was true for linoleic acid. Further trials should examine the potential role of linoleic acid and its main dietary source, vegetable oils, in abdominal obesity prevention.

## Introduction

Abdominal obesity (AO) is a strong predictor of cardiometabolic disease [1] and some cancer types[2, 3]. In particular, visceral obesity is a potential driver of insulin resistance and metabolic disorders[4]. The prevalence of AO is increasing [5, 6] and although genetic factors are influential, AO is largely determined by lifestyle [7]. Thus, lifestyle factors including diet might be key modifiable risk factors of AO[8, 9]. High intake of dietary fats has long been considered to promote AO, while recent studies suggest that the fatty acid (FA) compositing may be more important for modulating fat deposition and fat distribution [9, 10]. Diets high in saturated fatty acids (SFA) may promote the development of AO and metabolic syndrome [9–11], whereas high intake of polyunsaturated fatty acids (PUFA) may counteract body fat accumulation [9, 10]. In particular, linoleic acid (LA, 18:2n-6) and palmitic acid (PA, 16:0) have been reported to be associated with the degree of fat accumulation in both visceral and subcutaneous adipose tissue [9]. Apart from the potential role of dietary FA in AO, enzymes metabolizing FA may influence body fat storage, body weight[12], waist circumference, and obesity [13]. Stearoyl-CoA desaturase (SCD), Δ5-desaturase (D5D), and Δ6-desaturase (D6D) are together with elongases the main enzymes responsible for endogenous synthesis of monounsaturated FA and PUFA [14]. While SCD synthesizes monounsaturated FA from SFA, D5D and D6D catalyze the synthesis of long-chain PUFA, e.g., eicosapentaenoic acid (EPA) and docohexaenoic acid (DHA), from the two essential fatty acids, LA and α-linolenic acid (ALA).

Observational studies investigating relationships between fat intake and AO or related outcomes have mostly relied on self-estimated food intake, which may be limited by e.g. reporting bias and inaccuracy of food databases [15]. FA compositions in diverse physiological compartments, e.g., serum cholesteryl esters (CE), partly reflect FA composition of the diet [13], and are thus useful biomarkers of dietary fat quality [15]. Essential fatty acids (LA and ALA) and long-chain n-3 PUFA (EPA and DHA) are among the more reliable biomarkers of fat intake while many endogenously synthesized FA are often considered to reflect intake less correctly. However, intake of PA is at least partly reflected in serum CE. As men and women differ in abdominal fat accumulation as well as FA compositions in diet and tissues, it is possible that associations of serum FA and desaturase activities with measures of AO are sex-specific. In addition, altered desaturase activities (e.g. SCD) may be involved in pathophysiology of AO, but such mechanisms require further investigation [12, 13].

We hypothesized that serum FA composition, partly reflecting dietary fat quality, and desaturase activities are related to abdominal fat distribution, in a possible sex-dependent manner. We aimed to investigate cross-sectional associations of serum FA composition in CE and estimated desaturase activities with AO and anthropometric measures of abdominal adiposity in a large population-based cohort of 60-year-old Swedish men and women.

## Methods

### Study population

The cross-sectional study was conducted in a population-based cohort of 60-year-old men and women [16]. Data from baseline investigations were collected between August 1997 and March 1999. Every third man and woman living in the Stockholm County, Sweden, and born between July 1, 1937 and June 30, 1938 was invited to participate, i.e., a total of 5460 subjects (2779 men and 2681 women), of which 78% (n = 4232; 2039 men and 2193 women) agreed to take part [17, 18].

All subjects underwent a physical examination that included anthropometric measurements and blood sampling [16]. Blood samples were drawn in the morning after overnight fasting and serum glucose, insulin, cholesterol, and triglycerides were analyzed as previously described [16, 17]. A comprehensive questionnaire regarding dietary habits, lifestyle factors, and medical history was completed by all participants. Smoking, education and physical activity were categorized as described previously [18], while alcohol intake was estimated in g/day based on responses to five questions concerning intake of beer, wine, and spirits[19]. The study was approved by the Ethical Committee at Karolinska Institutet and all participants gave their informed verbal consent as previously described in detail [20]. Forms for written consent were not in current use and thus written consent was not collected. After having received written information about the study, those who decided to participate were asked to contact a booking central in order to make an appointment to attend a physical examination. The procedure for collecting verbal consent was approved by the Ethical Committee at Karolinska Institutet.

### Anthropometric measures

As previously described [17], body weight, height, sagittal abdominal diameter (SAD), waist circumference (WC), and hip circumference were recorded and utilized to calculate body mass index (BMI), sagittal abdominal diameter-to-height ratio (SADHR), waist-hip ratio (WHR), waist circumference-to-height ratio (WCHR), and waist-hip-height ratio (WHHR). The definition of the National Cholesterol Education Program (NCEP) Expert Panel on Detection, Evaluation, and Treatment of High Blood Cholesterol in Adults (Adult Treatment Panel III) was used for defining AO: WC>102 cm for men and WC>88 cm for women[21].

### Assessment of fatty acid composition

As previously described [18], FA composition in serum cholesteryl esters (CE) was measured by gas chromatography[22]. Proportions of individual FA were expressed as percentages of all measured FA. Desaturase activities were estimated as FA ratios and were calculated as follows: SCD = 16:1/16:0, D6D = 18:3n-6/18:2n-6, and D5D = 20:4n6/20:3n-6 [14].

### Statistical methods

Participants with missing data regarding exposures (FA and desaturase activities), AO measures (BMI, SAD, WC, WHR, SADHR, WCHR, WHHR), or covariates (physical activity, education, smoking and alcohol intake) were excluded before analyses (S1 Fig). Shapiro-Wilk’s test was performed to examine the normality of distribution for continuous variables. Sex differences of continuous variables were evaluated by Student’s *t*-test or Wilcoxon-Mann-Whitney test for normally and non-normally distributed variables, respectively. Differences between men and women in binary and ordinal variables were assessed by χ^2^-test.

Spearman’s rank correlation coefficients were calculated between FA (as well as desaturases) and anthropometric measurements. Crude and multivariable-adjusted logistic regression models were utilized to calculate odds ratio (OR) and 95% CI for prevalence of AO. Similarly, crude and multivariable-adjusted linear regression models were employed to investigate associations of FAs and estimated desaturase activities with abdominal anthropometric measures. All analyses were performed in the total study population and in sex-specific strata. Serum FA (EPA, DHA, LA, and ALA) were investigated as categorical (quartiles) variables and overall trends were evaluated with quartile medians as exposure. Restricted cubic splines were utilized for evaluation of potential nonlinear associations[18]. Physical activity, alcohol intake, education, and smoking were included as covariates in the adjusted models. In analyses on the total study population, sex was also included as a covariate and sex-differences in overall trends were evaluated by including an interaction term of sex and exposure (sex-specific quartile median) in the models. Statistical analyses were carried out with STATA version 13.0 (STATA Corporation, TX, USA). P-values < 0.05 were considered significant.

## Results

### General characteristics

After excluding participants with no data regarding serum FA, anthropometric measures, education, physical activity, alcohol intake and smoking habits, 3926 individuals (1899 men and 2027 women) of the cohort’s 4232 participants were included in the present study (S1 Fig). The proportion of individuals with abdominal obesity was greater (P<0.0001) among women (39%) compared to men (29%) (Table 1). Additional sex differences were found for all metabolic variables, degree of physical activity and smoking habits, where women were more sedentary and less likely to have been smokers than men. The correlation coefficients between the different anthropometric measures were between 0.50 and 0.96 (S1 Table).

**Table 1:** Anthropometrics, clinical measurements, lifestyle factors, serum cholesteryl ester FA, and estimated desaturase activity in the 60-year old men and women.

	Total (n = 3926)	Men (n = 1899)	Women (n = 2027)	*P*-value^1^
Abdominal obesity (%)	34	29	39	<0.0001
BMI (kg/m^2^)	26.3 (23.9–29.1)	26.6 (24.4–29.1)	25.9 (23.4–28.9)	<0.0001
SAD (cm)	20.6±2.9	21.5±2.7	19.8±2.8	<0.0001
Waist circumference (cm)	91.9±12.6	97.8±10.4	86.4±11.9	<0.0001
Waist-hip ratio	0.89±0.09	0.95±0.06	0.83±0.07	<0.0001
SADHR (%)	0.12±0.02	0.12±0.02	0.12±0.02	0.19
WCHR (%)	0.54±0.07	0.55±0.06	0.53±0.07	<0.0001
WHHR (m^-1^)	0.52±0.05	0.54±0.04	0.51±0.05	<0.0001
Total Cholesterol (mmol/L)	5.9 (5.3–6.6)	5.8 (5.1–6.4)	6.1 (5.4–6.8)	<0.0001
LDL Cholesterol^2^ (mmol/L)	3.9±0.9	3.8±0.9	3.9±1.0	0.019
HDL Cholesterol (mmol/L)	1.5±0.4	1.3±0.3	1.6±0.4	<0.0001
Triglycerides (mmol/L)	1.1 (0.8–1.6)	1.2 (0.9–1.7)	1.1 (0.8–1.5)	<0.0001
Fasting insulin (mU/L)	8.8 (6.6–12.3)	9.2 (6.8–13.4)	8.5 (6.4–11.5)	<0.0001
Fasting glucose^3^ (mmol/L)	5.2 (4.9–5.7)	5.4 (5.0–5.9)	5.1 (4.7–5.5)	<0.0001
Alcohol intake (g/day)	8.7 (2.7–17.6)	14.0 (6.7–26.5)	5.1 (1.3–11.6)	<0.0001
Physical activity (%)				<0.0001
Sedentary	11	11	12	
Light intensity	58	55	61	
Medium intensity	23	25	21	
High intensity	7	9	6	
Smoking (%)				<0.0001
Never	40	34	46	
Current	21	20	22	
Former	39	46	32	
Education (%)				0.11
≤9 y	28	27	30	
9–12 y	44	45	43	
>12 y	28	28	27	
Serum fatty acid, % of total FA				
Palmitic acid (PA, 16:0)	11.4 (10.9–11.9)	11.6 (11.1–12.0)	11.2 (10.8–11.6)	<0.0001
Linoleic acid (LA, 18:2n6)	48.4±4.2	48.4±4.2	48.4±4.1	0.97
Alpha-linolenic acid (ALA, 18:3n3)	0.88±0.20	0.87±0.20	0.90±0.20	<0.0001
Eicosapentaenoic acid (EPA, 20:5n3)	1.88 (1.46–2.47)	1.87 (1.44–2.46)	1.89 (1.47–2.49)	0.33
Docohexaenoic acid (DHA, 22:6n3)	0.92±0.25	0.91±0.25	0.93±0.24	0.0065
Desaturase activity, FA ratio				
SCD (16:1/16:0)	0.30 (0.24–0.38)	0.27 (0.22–0.35)	0.32 (0.27–0.40)	<0.0001
D5D (20:4n6/20:3n6)	9.0±2.2	9.0±2.2	9.0±2.2	0.41
D6D (18:3n6/18:2n6)	0.018 (0.013–0.023)	0.017 (0.013–0.023)	0.018 (0.014–0.024)	0.0059

Values are mean±SD, median (IQR), or %. D5D, Δ5-desaturase; D6D, Δ6-desaturase; FA, fatty acid; SAD, sagittal abdominal diameter; SADHR, sagittal abdominal diameter-to-height ratio; SCD, stearoyl-CoA desaturase; WCHR, waist circumference-to-height ratio; WHR, waist-hip ratio; WHHR, waist-hip-height ratio.

^1^Sex differences of continuous variables were assessed by Student’s *t*-test (normally distributed variables) or Wilcoxon-Mann-Whitney test (skewed variables), while sex difference in categorical variables were evaluated by χ^2^ test.

Proportions of ALA and DHA in serum CE as well as estimated activities of SCD and D6D were higher in women compared to men, who instead had greater proportion of serum PA (Table 1). PA correlated with the strongly intercorrelated EPA and DHA, while LA was inversely correlated with PA, EPA, and DHA (S1 Table). Serum ALA, correlated weakly with LA and EPA, and was inversely correlated with PA and DHA.

### Palmitic acid and abdominal obesity

**Fig 1:**
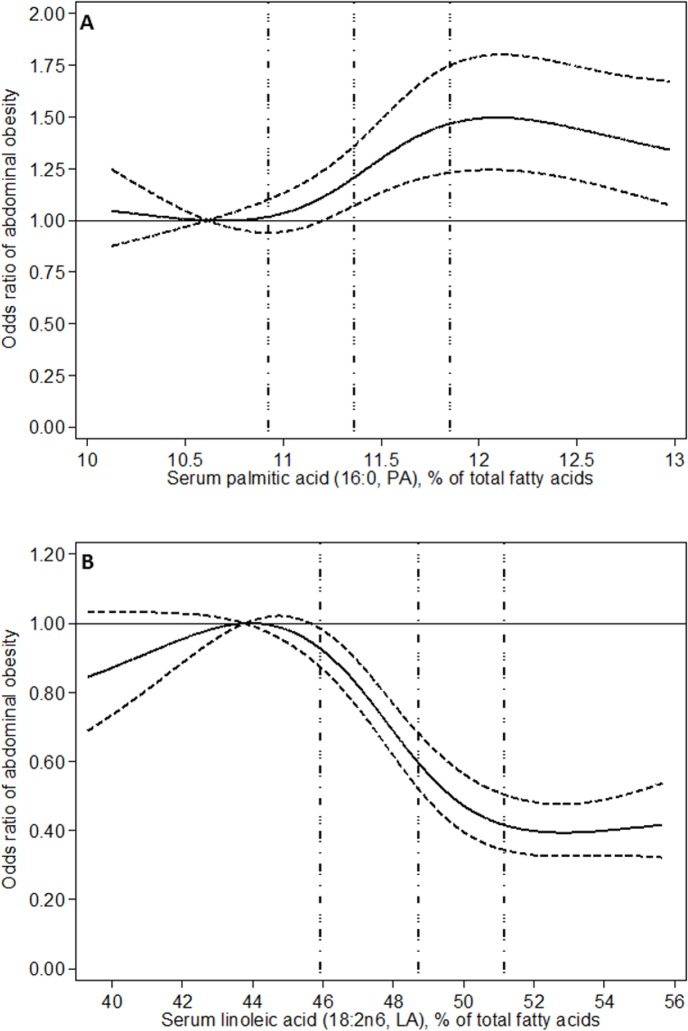
**Associations of serum palmitic acid (A) and linoleic acid (B) with abdominal obesity evaluated using restricted cubic spline.** Associations were adjusted for sex, smoking, physical activity, education, and alcohol intake. Full and dashed lines represent odds ratios and their 95% CI, respectively, while dotted vertical lines correspond to 25^th^, 50^th^, and 75^th^ percentiles of fatty acid proportions.

Serum PA was associated with AO (Table 2 and Fig 1A) with no significant (P = 0.11) difference between men and women (S2 Table). Comparing extreme quartiles of serum PA, the multivariable-adjusted odds of prevalent abdominal obesitywere 46% higher in the highest PA quartile (Table 2). Serum PA correlated with all anthropometric measures in men, but only to WHR in women (S1 Table). After adjustment for potential confounders, PA was in general associated with anthropometric measures (Table 3 and S3 Table). All associations of PA with AO and anthropometric measures were generally non-linear (Fig 1A, Table 2 and S2 and S3 Tables).

**Table 2:** Associations of serum fatty acids with abdominal obesity^1^.

			Quartile of serum fatty acid					
			1	2	3	4	P_trend_^2^	P_non-linear_^3^
*Palmitic acid*								
	Median (% of total FA)		10.6	11.2	11.6	12.2		
	AO prevalence^4^, n (%)		293 (30)	313 (32)	364 (37)	370 (38)		
	OR (95% CI)^5^							
	Sex-adjusted		1.00 (reference)	1.10 (0.91–1.34)	1.39 (1.15–1.68)	1.43 (1.19–1.73)	<0.0001	0.0097
	Mutivariable-adjusted^6^		1.00 (reference)	1.10 (0.90–1.33)	1.40 (1.15–1.70)	1.46 (1.20–1.77)	<0.0001	0.0037
*Linoleic acid*								
	Median (% of total FA)		43.8	47.4	49.9	52.9		
	AO prevalence^4^, n (%)		446 (45)	358 (36)	291 (30)	245 (25)		
	OR (95% CI)							
	Sex-adjusted		1.00 (reference)	0.69 (0.57–0.82)	0.50 (0.42–0.61)	0.40 (0.33–0.48)	<0.0001	<0.0001
	Mutivariable-adjusted		1.00 (reference)	0.69 (0.57–0.83)	0.50 (0.41–0.61)	0.40 (0.32–0.49)	<0.0001	<0.0001
*Alpha-linoleic acid*								
	Median (% of total FA)		0.67	0.81	0.93	1.10		
	AO prevalence^4^, n (%)		376 (38)	333 (34)	324 (33)	307 (31)		
	OR (95% CI)							
	Sex-adjusted		1.00 (reference)	0.83 (0.69–0.99)	0.79 (0.66–0.95)	0.73 (0.61–0.88)	0.0013	0.07
	Mutivariable-adjusted		1.00 (reference)	0.87 (0.72–1.05)	0.80 (0.67–0.97)	0.74 (0.61–0.89)	0.0014	0.10
*Eicosapentaenoic acid*								
	Median (% of total FA)		1.20	1.66	2.13	3.08		
	AO prevalence^4^, n (%)		322 (33)	361 (37)	340 (35)	317 (32)		
	OR (95% CI)							
	Sex-adjusted		1.00 (reference)	1.19 (0.99–1.44)	1.09 (0.90–1.31)	0.98 (0.81–1.18)	0.39	0.32
	Mutivariable-adjusted		1.00 (reference)	1.24 (1.02–1.50)	1.19 (0.98–1.44)	1.09 (0.90–1.33)	0.75	0.13
*Docohexaenoic acid*								
	Median (% of total FA)		0.65	0.82	0.97	1.19		
	AO prevalence^4^, n (%)		374 (38)	345 (35)	343 (35)	278 (28)		
	OR (95% CI)							
	Sex-adjusted		1.00 (reference)	0.88 (0.73–1.06)	0.87 (0.72–1.05)	0.64 (0.53–0.77)	<0.0001	0.34
	Mutivariable-adjusted		1.00 (reference)	0.94 (0.78–1.14)	0.97 (0.80–1.17)	0.75 (0.62–0.92)	0.0088	0.18

^1^FA, fatty acid; OR, odds ratio; SAD, sagittal abdominal diameter; WC, waist circumference; WHR, waist-hip ratio.

^2^P for overall trend (P_trend_) was evaluated using logistic regression models with sex-specific quartile median as exposure.

^3^P for nonlinearity (P_non-linear_) was evaluated using restricted cubic splines.

^4^Abdominal obesity was defined as WC>88 cm in women and WC>102 cm in men.

^5^OR and 95% CI were evaluated using logistic regression models.

^6^Adjusted for sex, physical activity, alcohol intake, education and smoking.

**Table 3:** Associations of serum fatty acids with abdominal obesity^1^^,^^2^.

			Quartile of serum fatty acid					
			1	2	3	4	P_trend_^3^	P_non-linear_^4^
*Palmitic acid*								
	SAD, cm	Observed	20.3 (20.2–20.5)	20.5 (20.3–20.6)	20.7 (20.5–20.9)	20.9 (20.7–21.0)	<0.0001	0.005
		Adjusted^5^	20.3 (20.2–20.5)	20.5 (20.3–20.6)	20.7 (20.5–20.9)	20.9 (20.7–21.0)	<0.0001	0.002
	WC, cm	Observed	90.6 (89.8–91.4)	91.3 (90.6–92.1)	92.5 (91.7–93.3)	93.2 (92.4–94.0)	<0.0001	0.0031
		Adjusted	90.6 (89.9–91.3)	91.3 (90.6–92.0)	92.5 (91.8–93.2)	93.2 (92.6–93.9)	<0.0001	0.0010
	WHR, cm	Observed	0.88 (0.87–0.88)	0.88 (0.88–0.89)	0.89 (0.89–0.90)	0.90 (0.89–0.90)	<0.0001	0.0023
		Adjusted	0.88 (0.88–0.88)	0.88 (0.88–0.89)	0.89 (0.89–0.89)	0.90 (0.89–0.90)	<0.0001	0.0008
*Linoleic acid*								
	SAD, cm	Observed	21.3 (21.1–21.5)	20.9 (20.7–21.1)	20.3 (20.1–20.5)	19.9 (19.7–20.0)	<0.0001	<0.0001
		Adjusted	21.3 (21.1–21.5)	20.9 (20.7–21.0)	20.3 (20.2–20.5)	19.9 (19.7–20.0)	<0.0001	<0.0001
	WC, cm	Observed	94.9 (94.1–95.7)	93.0 (92.2–93.8)	90.8 (90.1–91.6)	88.9 (88.2–89.7)	<0.0001	<0.0001
		Adjusted	94.9 (94.2–95.6)	93.0 (92.3–93.6)	90.8 (90.1–91.5)	88.9 (88.2–89.6)	<0.0001	<0.0001
	WHR, cm	Observed	0.91 (0.90–0.91)	0.89 (0.89–0.90)	0.88 (0.88–0.89)	0.87 (0.87–0.88)	<0.0001	0.0007
		Adjusted	0.91 (0.90–0.91)	0.89 (0.89–0.89)	0.88 (0.88–0.89)	0.87 (0.87–0.88)	<0.0001	0.0011
*Alpha-linoleic acid*								
	SAD, cm	Observed	20.8 (20.6–21.0)	20.6 (20.4–20.8)	20.5 (20.3–20.7)	20.4 (20.3–20.6)	0.0011	0.21
		Adjusted	20.8 (20.6–21.0)	20.6 (20.5–20.8)	20.5 (20.3–20.7)	20.4 (20.2–20.6)	0.0012	0.42
	WC, cm	Observed	92.9 (92.1–93.7)	91.8 (91.0–92.6)	91.5 (90.7–92.3)	91.4 (90.7–92.1)	0.0032	0.08
		Adjusted	92.8 (92.1–93.4)	92.0 (91.3–92.7)	91.5 (90.8–92.2)	91.4 (90.7–92.1)	0.004	0.17
	WHR, cm	Observed	0.89 (0.89–0.90)	0.89 (0.88–0.89)	0.89 (0.88–0.89)	0.89 (0.88–0.89)	0.51	0.0062
		Adjusted	0.89 (0.89–0.89)	0.89 (0.88–0.89)	0.89 (0.88–0.89)	0.89 (0.88–0.89)	0.50	0.019
*Eicosapentaenoic acid*								
	SAD, cm	Observed	20.4 (20.2–20.6)	20.7 (20.5–20.9)	20.7 (20.5–20.8)	20.6 (20.4–20.7)	0.62	0.0354
		Adjusted	20.4 (20.2–20.5)	20.7 (20.5–20.8)	20.7 (20.5–20.9)	20.6 (20.4–20.8)	0.13	0.0076
	WC, cm	Observed	91.4 (90.6–92.2)	92.3 (91.5–93.0)	92.2 (91.4–93.0)	91.8 (91.0–92.6)	0.71	0.11
		Adjusted	91.1 (90.4–91.8)	92.1 (91.4–92.8)	92.3 (91.7–93.0)	92.1 (91.4–92.8)	0.11	0.0203
	WHR, cm	Observed	0.89 (0.88–0.89)	0.89 (0.89–0.90)	0.89 (0.88–0.89)	0.88 (0.88–0.89)	0.10	0.42
		Adjusted	0.89 (0.88–0.89)	0.89 (0.89–0.89)	0.89 (0.88–0.89)	0.89 (0.88–0.89)	0.61	0.57
*Docohexaenoic acid*								
	SAD, cm	Observed	20.8 (20.6–21.0)	20.6 (20.4–20.8)	20.6 (20.4–20.8)	20.4 (20.2–20.6)	0.0016	0.31
		Adjusted	20.7 (20.5–20.8)	20.6 (20.4–20.8)	20.6 (20.4–20.8)	20.5 (20.3–20.7)	0.18	0.17
	WC, cm	Observed	92.5 (91.8–93.3)	92.1 (91.3–92.9)	91.8 (91.0–92.7)	91.2 (90.4–91.9)	0.0080	0.30
		Adjusted	92.1 (91.4–92.8)	92.0 (91.3–92.7)	91.9 (91.3–92.6)	91.6 (90.9–92.3)	0.45	0.14
	WHR, cm	Observed	0.89 (0.89–0.90)	0.89 (0.88–0.89)	0.89 (0.88–0.89)	0.88 (0.88–0.89)	<0.0001	0.61
		Adjusted	0.89 (0.89–0.90)	0.89 (0.88–0.89)	0.89 (0.88–0.89)	0.88 (0.88–0.89)	0.0144	0.91

^1^FA, fatty acid; SAD, sagittal abdominal diameter; WC, waist circumference; WHR, waist-hip ratio.

^2^Values are quartile means (95% CI).

^3^P for overall trend (P_trend_) was evaluated using linear regression models with sex-specific quartile median as exposure; for observed values sex was the only additional covariate, while for multivariable-adjusted trend sex, physical activity, alcohol intake, education and smoking were included as covariates.

^4^P for nonlinearity (P_non-linear_) was evaluated using restricted cubic splines

^5^Adjusted for physical activity, alcohol intake, education and smoking.

### Linoleic acid and abdominal obesity

The prevalence of AO was significantly lower with higher LA levels (Table 2 and Fig 1B), with no difference between men and women (P = 0.53) (S2 Table). The multivariable-adjusted odds of prevalent abdominal obesity were 60% lower in the highest compared to lowest LA quartile (Table 2). Serum LA was negatively correlated to all anthropometric measures in both sexes (S1 Table) and the inverse associations between LA and anthropometric measures remained after adjusting for potential confounders in men and women, separately (S3 Table) as well as combined (Table 3). Associations of LA with AO and anthropometric measures did not differ between men and women and were generally non-linear (Fig 1B, Table 2 and S2 Table).

### n-3 PUFA and abdominal obesity

The prevalence of AO was lower with higher levels of serum ALA (Table 2). However, there was a significant sex-difference (P = 0.0017), where an inverse association between ALA and AO was only observed in men when sexes were evaluated separately (S2 Table). Serum ALA was also negatively correlated with all anthropometric measures in men, but generally not in women (S1 Table). Results were similar after adjusting for potential confounders (S3 Table). In the total study population, ALA was inversely associated with SAD and WC but not with WHR (Table 3).

Serum EPA was not associated with AO and did not associate with any anthropometric measures in the total study population (Tables 2 and 3). However, there was a borderline significant sex-difference (P = 0.05) in the association between EPA and AO; and EPA was associated with AO (P = 0.03), SAD (P = 0.03), and WC (P = 0.02) in women, when evaluated in sex-specific analyses using multivariable-adjusted models (S2 and S3 Tables).

Serum DHA was inversely associated with AO prevalence (Table 2), with no significant sex-differences (P = 0.23). When stratified by sex, however, an inverse association between DHA and AO was only observed in women (S2 Table). In addition, all anthropometric measures were inversely correlated with DHA among women (S1 Table), but after adjustment for potential confounders, DHA was inversely associated only with WHR, in the total study population (Table 3) and in women (S3 Table).

The associations between n-3 PUFA and AO and anthropometric measures, respectively, generally appeared to be linear (Tables 2 and 3).

### Desaturase activities and abdominal obesity

The estimated activities of SCD and D6D were associated with AO (Table 4), with no significant (P≥0.09) sex-differences (S4 Table). Both SCD and D6D activities were correlated (S1 Table) and associated with all anthropometric measures (Table 5), regardless of sex (S5 Table).

**Table 4:** Associations of estimated desaturase activities with abdominal obesity^1^.

		Quartile of estimated desaturase activity					
		1	2	3	4	P_trend_^2^	P_non-linear_^3^
*SCD*							
	Median 16:1/16:0 ratio	0.21	0.27	0.33	0.45		
	AO prevalence^4^, n (%)	190 (19)	269 (27)	405 (41)	476 (49)		
	OR (95% CI)^5^						
	Sex-adjusted	1.00 (reference)	1.58 (1.28–1.95)	2.96 (2.42–3.63)	4.00 (3.27–4.91)	<0.0001	<0.0001
	Multivariable-adjusted^6^	1.00 (reference)	1.61 (1.29–1.99)	3.01 (2.44–3.72)	4.14 (3.33–5.15)	<0.0001	<0.0001
*D5D*							
	Median 20:4n6/20:3n6 ratio	6.71	8.11	9.49	11.46		
	AO prevalence, n (%)	431 (44)	385 (39)	289 (29)	235 (24)		
	OR (95% CI)						
	Sex-adjusted	1.00 (reference)	0.82 (0.69–0.99)	0.53 (0.44–0.64)	0.40 (0.33–0.48)	<0.0001	0.0004
	Multivariable-adjusted	1.00 (reference)	0.84 (0.70–1.01)	0.53 (0.44–0.64)	0.40 (0.33–0.49)	<0.0001	0.0005
*D6D*							
	Median 18:2n6/18:3n6 ratio	0.011	0.016	0.020	0.028		
	AO prevalence, n (%)	231 (24)	271 (28)	356 (36)	482 (49)		
	OR (95% CI)						
	Sex-adjusted	1.00 (reference)	1.24 (1.01–1.52)	1.86 (1.53–2.27)	3.19 (2.63–3.89)	<0.0001	0.0021
	Multivariable-adjusted	1.00 (reference)	1.19 (0.97–1.47)	1.78 (1.45–2.17)	3.00 (2.45–3.66)	<0.0001	0.0038

^1^D5D, Δ5-desaturase; D6D, Δ6-desaturase; SAD, sagittal abdominal diameter; SCD, stearoyl-CoA desaturase WC, waist circumference; WHR, waist-hip ratio.

^2^P for overall trend (P_trend_) was evaluated using logistic regression models with sex-specific quartile median as exposure.

^3^P for nonlinearity (P_non-linear_) was evaluated using restricted cubic splines.

^4^Abdominal obesity was defined as WC>88 cm in women and WC>102 cm in men.

^5^OR and 95% CI were evaluated using logistic regression models.

^6^Adjusted for sex, physical activity, alcohol intake, education and smoking.

**Table 5:** Associations of estimated desaturase activities with anthropometric measures.^1^^,^^2^.

			Quartile of estimated desaturase activity					
			1	2	3	4	P_trend_^3^	P_non-linear_^4^
*SCD*								
	SAD, cm	Observed	19.5 (19.4–19.7)	20.2 (20.0–20.3)	21.0 (20.9–21.2)	21.6 (21.4–21.8)	<0.0001	<0.0001
		Adjusted^5^	19.5 (19.4–19.7)	20.2 (20.0–20.3)	21.0 (20.9–21.2)	21.6 (21.5–21.8)	<0.0001	<0.0001
	WC, cm	Observed	87.4 (86.7–88.2)	90.5 (89.8–91.2)	93.9 (93.1–94.7)	95.8 (95.0–96.6)	<0.0001	<0.0001
		Adjusted	87.4 (86.7–88.1)	90.5 (89.8–91.2)	93.9 (93.2–94.5)	95.9 (95.2–96.6)	<0.0001	<0.0001
	WHR, cm	Observed	0.87 (0.86–0.87)	0.88 (0.88–0.89)	0.89 (0.89–0.90)	0.91 (0.90–0.91)	<0.0001	<0.0001
		Adjusted	0.87 (0.86–0.87)	0.88 (0.88–0.88)	0.89 (0.89–0.90)	0.91 (0.90–0.91)	<0.0001	<0.0001
*D5D*								
	SAD, cm	Observed	21.4 (21.2–21.6)	20.9 (20.7–21.0)	20.3 (20.1–20.5)	19.8 (19.6–20.0)	<0.0001	<0.0001
		Adjusted	21.4 (21.2–21.6)	20.9 (20.7–21.0)	20.3 (20.1–20.5)	19.8 (19.6–20.0)	<0.0001	<0.0001
	WC, cm	Observed	95.2 (94.5–96.0)	92.9 (92.1–93.7)	90.7 (89.9–91.5)	88.8 (88.0–89.5)	<0.0001	<0.0001
		Adjusted	95.1 (94.4–95.8)	92.9 (92.3–93.6)	90.7 (90.1–91.4)	88.8 (88.2–89.5)	<0.0001	<0.0001
	WHR, cm	Observed	0.90 (0.90–0.91)	0.89 (0.89–0.90)	0.88 (0.88–0.89)	0.87 (0.87–0.88)	<0.0001	0.0005
		Adjusted	0.90 (0.90–0.91)	0.89 (0.89–0.90)	0.88 (0.88–0.89)	0.87 (0.87–0.88)	<0.0001	0.0005
*D6D*								
	SAD, cm	Observed	19.7 (19.6–19.9)	20.2 (20.1–20.4)	20.8 (20.6–21.0)	21.6 (21.4–21.8)	<0.0001	<0.0001
		Adjusted	19.8 (19.6–20.0)	20.2 (20.1–20.4)	20.8 (20.6–21.0)	21.5 (21.4–21.7)	<0.0001	0.0001
	WC, cm	Observed	88.4 (87.6–89.1)	90.5 (89.7–91.3)	93.0 (92.2–93.7)	95.8 (95.0–96.5)	<0.0001	<0.0001
		Adjusted	88.6 (88.0–89.3)	90.5 (89.9–91.2)	92.9 (92.2–93.6)	95.5 (94.9–96.2)	<0.0001	<0.0001
	WHR, cm	Observed	0.87 (0.86–0.87)	0.88 (0.87–0.89)	0.89 (0.89–0.90)	0.91 (0.90–0.91)	<0.0001	<0.0001
		Adjusted	0.87 (0.87–0.88)	0.88 (0.88–0.88)	0.89 (0.89–0.90)	0.90 (0.90–0.91)	<0.0001	<0.0001

^1^D5D, Δ5-desaturase; D6D, Δ6-desaturase; SAD, sagittal abdominal diameter; SCD, stearoyl-CoA desaturase; WC, waist circumference; WHR, waist-hip ratio.

^2^Values are quartile means (95% CI).

^3^P for overall trend (P_trend_) was evaluated linear regression models with sex-specific quartile median as exposure; for observed values sex was the only additional covariate, while for multivariable-adjusted trend sex,physical activity, alcohol intake, education and smoking were included as covariates.

^4^P for nonlinearity (P_non-linear_) was evaluated using restricted cubic splines.

^5^Values were adjusted for sex, physical activity, alcohol intake, education and smoking.

The estimated activity of D5D was inversely associated with AO (Table 4) and the association did not differ (P = 0.61) between men and women (S4 Table). Similarly, D5D was negatively correlated (S1 Table) and inversely associated with all anthropometric measures (Table 5 and S5 Table). The inverse association of D5D with WHR was somewhat stronger in women (P<0.05). Associations between desaturase activities and AO and anthropometric measures were generally non-linear (Tables 4 and 5).

In general, associations of fatty acids and desaturase activities with the novel anthropometric measures (SADHR, WCHR, and WHHR) were similar to the associations with the more traditional measures SAD, WC, and WHHR (data not shown).

## Discussion

The present study represents the largest cross-sectional study to date investigating the relationships between serum FA as biomarkers of dietary fat quality and AO in men and women. In line with current dietary guidelines, the results suggest that higher intake of PUFA, n-6 in particular, is associated with lower AO. A higher proportion of PA in serum was associated with higher prevalence of AO, whilst the contrary was found for serum LA. Associations between n-3 PUFA and AO were in general weaker and partly sex-specific.

The positive and inverse associations with AO of PA and LA, respectively, are supported by previous findings in observational studies and clinical trials. Serum PA has been correlated to measures of AO [13, 23] and in addition, PA in other compartments (i.e., plasma, erythrocytes, and skeletal muscle phospholipids) has been associated with increased liver fat [24], body fat percentage [25], and BMI [25]. In line with the present findings, high serum proportions of LA have been associated with lower SAD [13], WC [13], WHR [23], and BMI [13, 23]. When relations of serum PUFA with all-cause mortality and incident CVD were evaluated in the present study population, LA was inversely associated with all-cause mortality but not with CVD risk[18]. A recent meta-analysis reported lower LA in plasma phospholipids among overweight compared to normal weight participants [26] and LA concentration in LDL phosphatidylcholine has been associated with lower BMI and WC [27]. Findings from observational studies are supported by randomized controlled trials reporting greater accumulation of liver fat [9, 10], visceral fat [9], and total body fat [9] after consumption of SFA (high in PA) compared to PUFA (high in LA). The different effects on body composition by PUFA and SFA consumption may be partly due to PUFA-induced inhibition of *de novo* lipogenesis [9, 10]. Serum PA proportions are however not exclusively determined by PA intake, but also by endogenous FA metabolism (e.g., PA can be synthesized from especially sugars and other refined carbohydrates through *de novo* lipogenesis, and PA may undergo elongation or desaturation). However, the participants in the present study likely consumed a rather non-lipogenic diet with limited consumption of sugar-sweetened beverages and generally high (>30%) fat intake [28]. Other mechanisms behind the current associations may include greater oxidation of dietary PUFA versus SFA [29] or a potential obesogenic effect of SFA *per se* by up-regulation of 11β-hydroxysteroid-dehydrogenase type 1, promoting cortisol induced visceral fat accumulation [30].

In line with previous findings [23], serum ALA was inversely associated with measures of AO in the present study. When stratified by sex, associations were only observed in men, maybe due to a weaker relationship between ALA intake and serum levels in women as we speculated earlier [18, 31].

In the present study population, long-chain n3 PUFA in serum were associated with lower risk of incident CVD and all-cause mortality in a partly sex-dependent manner [18]. Here, serum DHA was inversely associated with AO in the total study population and in women but not in men. A recent meta-analysis reported lower plasma DHA in overweight compared to normal weight participants [26]. Effects on fat distribution by DHA *per se* may be limited and sex-specific associations of serum n3-PUFA and AO could partly reflect different dietary and lifestyle patterns in men and women. However, adjustments for lifestyle factors did not completely attenuate the associations between DHA and AO in the present study. Furthermore, dissimilarities in associations between men and women could be due to general differences in fat accumulation [32] or by hormone-dependent sex differences in lipid metabolism as suggested by human tracer trials [33], animal studies [34], and in vitro experiments [34].

Similar to the present study, activities of SCD and D6D have been associated with AO [13] and subcutaneous adipose tissue [35]. Higher SCD activity has also been observed in individuals with increased liver fat content [24]. On the contrary, D5D activity estimated in plasma compartments has been inversely associated with AO [13] and subcutaneous adipose tissue [35]. In a recent meta-analysis, overweight individuals had higher D6D, but lower D5D activity estimates than those with normal weight [26]. Whether estimated desaturase activity affects body composition or rather is a marker of lifestyle and diet quality remains to be determined [36]. However, evidence of relationships between desaturases and body composition are supported by studies reporting associations between genetic variation of SCD and waist circumference [37] as well as genome-wide associations between D5D- and D6D-encoding genes and appendicular lean mass [38]. Considering the markedly elevated risk of OA in subjects with high estimated SCD activity, this finding warrants further research to examine if SCD activity is an important interventional target to reduce or prevent AO in humans.

Some limitations of the present study should be highlighted. Results from this cross-sectional study support findings from randomized clinical trials and previous observational studies, but no inference of causation can be made from the current results due to the cross-sectional study design. The present cohort is restricted to 60-year-olds in Stockholm County and thus the results may not be representative for other populations. Assessments of serum FA and measures of AO were only performed once, which may lead to misclassifications due to intra-individual variation. However, the seemingly good reproducibility of serum PUFA [39] and anthropometry [40] as well as the consistency of results for different anthropometric measures suggest that these are not chance findings. Estimated desaturase activities may imperfectly reflect the *in vivo* desaturase activities although good agreement between FA ratios and more directly measured activity has been reported previously [41]. As FA proportions were utilized in the present study, it cannot be concluded that associations of single FA were independent of other FA (e.g., whether AO is associated with low LA consumption or high intake of PA and/or PA precursors). However, findings from trials suggest that dietary LA more effectively prevents abdominal fat accumulation compared to PA [9, 10]. In the present study, fatty acid composition was assessed in cholesterol esters and it cannot be excluded that the results would have been slightly different if we used compartment, e.g. phospholipids. Although fatty acid proportions in cholesterol esters and phospholipids generally correlate strongly [42], phospholipids comprise a larger fatty acid pool and proportions of palmitic acid in particular do differ between these two circulating compartments. Furthermore, it can be argued to what degree the anthropometric measures utilized distinguish between different types of abdominal fat (e.g., subcutaneous and visceral). Finally, residual confounding cannot be excluded.

A major strength of the study is the use of a large population-based cohort with high participation rate assessing serum FA composition. Biomarkers can provide a more objective estimate of dietary fat composition compared to traditional assessment methods based on self-reports. Associations of serum FA and desaturases with AO were evaluated by calculating odds ratios of AO defined by common thresholds as well as by assessing linear and nonlinear relationships with several anthropometric measures of AO. Finally, the inclusion of both men and women allowed investigations of sex-specific relationships.

## Conclusion

Serum proportions of fatty acids, partly reflecting dietary fat intake, were associated with abdominal obesity in this large-scale population-based study and the associations were to some extent sex-specific. The most abundant serum fatty acid, linoleic acid, was strongly and inversely associated with abdominal obesity in both men and women. Contrary, a high serum proportion of palmitic acid, a major saturated fatty acid, was linked to higher odds of abdominal obesity and greater levels of all anthropometric measures. Docohexaenoic acid and α-linoleic acid were inversely associated with AO, in a partly sex-specific manner. Overall, these findings support those of recent interventional and experimental studies suggesting that a higher relative intake of polyunsaturated fatty acids (especially linoleic acid) from vegetable oils, associates with decreased abdominal adiposity. These findings are therefore coherent with current dietary guidelines regarding partial replacement of saturated fats with polyunsaturated fatty acids, especially in the light of the high and increasing prevalence of abdominal obesity and related diseases (e.g., diabetes and cardiovascular disease). In accordance with previous studies, fatty acid desaturase activities were altered in people with abdominal obesity.

## Supporting Information

S1 Fig Participant flow chart. Participants with no missing data regarding exposures (serum fatty acid and desaturase activities), outcomes (abdominal obesity measures), or covariates (physical activity, education, smoking and alcohol intake) were included for statistical analysis.
(PDF)

S1 Table Spearman’s rank correlation coefficients between anthropometric measurements, serum fatty acids, and estimated desaturase activities. (PDF)

S2 Table Associations of serum fatty acids with abdominal obesity in men and women. (PDF)

S3 Table Associations of serum fatty acids with anthropometric measures in men and women. (PDF)

S4 Table Associations of estimated desaturase activities with abdominal obesity. (PDF)

S5 Table Associations of estimated desaturase activities with anthropometric measures in men and women. (PDF)
