# Supplementary material for: Serum Fatty Acids, Desaturase Activities and Abdominal Obesity – A Population-Based Study of 60-Year Old Men and Women
Source: PLoS One. 2017 Jan 26;12(1):e0170684. doi: 10.1371/journal.pone.0170684 (PMC5270324; doi:10.1371/journal.pone.0170684)
Supplement: S1 Fig — Participants with no missing data regarding exposures (serum fatty acid and desaturase activities), outcomes (abdominal obesity measures), or covariates (physical activity, education, smoking and alcohol intake) were included for statistical analysis. (PDF) [file pone.0170684.s001.pdf]

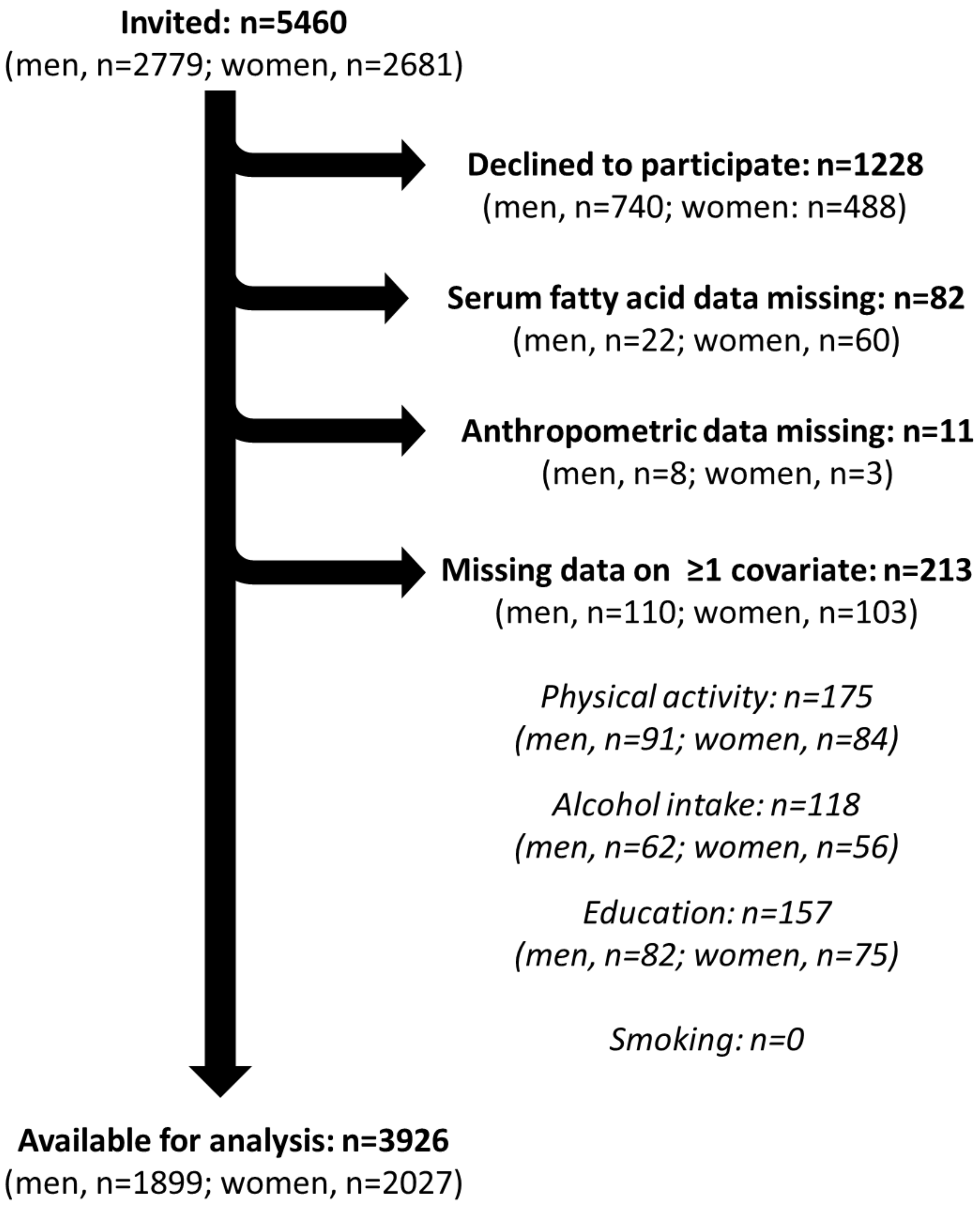

Fig S1. Participant flow chart. Participants with no missing data regarding exposures (serum fatty acid and desaturase activities), outcomes (abdominal obesity measures), or covariates (physical activity, education, smoking and alcohol intake) were included for statistical analysis.
