# Supplementary material for: Serum Fatty Acids, Desaturase Activities and Abdominal Obesity – A Population-Based Study of 60-Year Old Men and Women
Source: PLoS One. 2017 Jan 26;12(1):e0170684. doi: 10.1371/journal.pone.0170684 (PMC5270324; doi:10.1371/journal.pone.0170684)
Supplement: S1 Table — (PDF) [file pone.0170684.s002.pdf]

S1 Table. Spearman's rank correlation coefficients between anthropometric measurements, serum fatty acids, and estimated desaturase activities.[1,2]

Men/Women[1]	BMI	SAD	WC	WHR	SADHR	WCHR	WHHR	PA	LA	ALA	EPA	DHA	SCD	D5D	D6D
BMI		0.83	0.86	0.50	0.85	0.88	0.50	0.02	-0.14	-0.06	0.01	-0.05	0.26	-0.22	0.25
SAD	0.83		0.87	0.59	0.96	0.84	0.50	0.00	-0.16	-0.03	0.00	-0.10	0.29	-0.25	0.27
WC	0.88	0.87		0.78	0.83	0.96	0.67	0.04	-0.19	-0.01	0.00	-0.09	0.30	-0.26	0.29
WHR	0.63	0.71	0.82		0.59	0.79	0.90	0.07	-0.19	0.04	-0.03	-0.11	0.25	-0.22	0.26
SADHR	0.84	0.95	0.81	0.71		0.88	0.62	-0.02	-0.15	-0.05	-0.02	-0.10	0.28	-0.25	0.27
WCHR	0.90	0.83	0.93	0.83	0.88		0.78	0.02	-0.18	-0.03	-0.02	-0.11	0.30	-0.26	0.29
WHHR	0.55	0.52	0.57	0.84	0.68	0.78		0.03	-0.15	0.00	-0.06	-0.12	0.23	-0.21	0.24
PA	0.14	0.15	0.17	0.15	0.12	0.14	0.08		-0.69	-0.17	0.37	0.38	0.21	0.19	0.19
LA	-0.19	-0.24	-0.23	-0.23	-0.22	-0.21	-0.16	-0.68		0.05	-0.40	-0.22	-0.64	-0.11	-0.59
ALA	-0.11	-0.07	-0.10	-0.08	-0.09	-0.12	-0.10	-0.16	0.05		0.07	-0.17	0.14	-0.26	0.05
EPA	0.00	0.01	0.01	-0.03	-0.03	-0.03	-0.10	0.38	-0.43	0.10		0.67	0.00	0.37	0.09
DHA	0.00	-0.01	0.00	-0.04	-0.04	-0.03	-0.09	0.35	-0.21	-0.13	0.72		-0.19	0.36	-0.23
SCD	0.27	0.32	0.28	0.27	0.32	0.29	0.23	0.23	-0.66	0.11	0.07	-0.18		-0.30	0.59
D5D	-0.23	-0.24	-0.21	-0.15	-0.25	-0.22	-0.14	0.10	-0.12	-0.20	0.32	0.29	-0.23		-0.14
D6D	0.23	0.26	0.24	0.23	0.26	0.25	0.19	0.32	-0.66	-0.04	0.14	-0.17	0.62	-0.10	

ALA, α-linolenic acid; D5D, Δ5-desaturase; D6D, Δ6-desaturase; EPA, eicosapentaenoic acid; DHA, docohexaenoic acid; LA, linoleic acid; PA, palmitic acid; SAD, sagittal abdominal diameter; SADHR, sagittal abdominal diameter-to-height ratio; SCD, steaoryl-CoA-desaturase; WC, waist circumference; WCHR, waist circumference-to-height ratio; WHR, waist-hip ratio; WHHR, waist-hip-height ratio.

[1]Spearman's rank correlation coefficients (r) for women and men are shown above and below the diagonal, respectively.

[2]Significance of r: $|r| > 0.04$, $P<0.05$; $|r| \geq 0.06$, $P<0.01$; $|r| \geq 0.07$ in women or $|r| \geq 0.08$ in men, $P<0.001$; $|r| > 0.09$, $P<0.0001$.
