# Supplementary material for: Serum Fatty Acids, Desaturase Activities and Abdominal Obesity – A Population-Based Study of 60-Year Old Men and Women
Source: PLoS One. 2017 Jan 26;12(1):e0170684. doi: 10.1371/journal.pone.0170684 (PMC5270324; doi:10.1371/journal.pone.0170684)
Supplement: S2 Table — (PDF) [file pone.0170684.s003.pdf]

S2 Table. Associations of serum fatty acids with abdominal obesity in men and women[1]

		Quartile of serum fatty acids					
		1	2	3	4	P_{trend}[2]	$P_{non-linear}$[3]
Palmitic acid (16:0, PA)							
Men	Median (% of total FA)	10.8	11.3	11.8	12.4		
	AO prevalence[4], n (%)	114 (24)	115 (24)	157 (33)	163 (34)		
	Crude OR[5] (95% CI)	1.00 (reference)	1.01 (0.75-1.36)	1.56 (1.18-2.08)	1.66 (1.25-2.20)	<0.0001	0.0093
	Adjusted OR[5,6] (95% CI)[6]	1.00 (reference)	0.97 (0.72-1.32)	1.58 (1.18-2.12)	1.61 (1.20-2.16)	0.0001	0.0090
Women	Median (% of total FA)	10.5	11.0	11.4	12.0		
	AO prevalence[4], n (%)	179 (35)	198 (39)	207 (41)	207 (41)		
	Crude OR[5] (95% CI)	1.00 (reference)	1.17 (0.91-1.52)	1.26 (0.98-1.63)	1.27 (0.98-1.64)	0.06	0.06
	Adjusted OR[5,6] (95% CI)[6]	1.00 (reference)	1.20 (0.93-1.56)	1.30 (1.00-1.69)	1.39 (1.07-1.80)	0.0141	0.0296
Linoleic acid (18:2n6, LA)							
Men	Median (% of total FA)	43.7	47.4	50.0	52.9		
	AO prevalence[4], n (%)	181 (38)	149 (31)	119 (25)	100 (21)		
	Crude OR[5] (95% CI)	1.00 (reference)	0.74 (0.57-0.97)	0.54 (0.41-0.72)	0.43 (0.33-0.58)	<0.0001	<0.0001
	Adjusted OR[5,6] (95% CI)[6]	1.00 (reference)	0.78 (0.59-1.04)	0.57 (0.42-0.76)	0.47 (0.34-0.64)	<0.0001	<0.0001
Women	Median (% of total FA)	43.8	47.3	49.8	52.8		
	AO prevalence[4], n (%)	265 (52)	209 (41)	172 (34)	145 (29)		
	Crude OR[5] (95% CI)	1.00 (reference)	0.64 (0.50-0.82)	0.47 (0.36-0.60)	0.37 (0.28-0.48)	<0.0001	0.0061
	Adjusted OR[5,6] (95% CI)[6]	1.00 (reference)	0.62 (0.48-0.80)	0.45 (0.35-0.59)	0.34 (0.26-0.45)	<0.0001	0.0132
α-linolenic acid							
Men	Median (% of total FA)	0.66	0.80	0.91	1.09		
	AO prevalence[4], n (%)	169 (36)	137 (29)	128 (27)	115 (24)		
	Crude OR[5] (95% CI)	1.00 (reference)	0.73 (0.56-0.96)	0.67 (0.51-0.88)	0.58 (0.44-0.77)	0.0001	0.20
	Adjusted OR[5,6] (95% CI)[6]	1.00 (reference)	0.77 (0.59-1.02)	0.67 (0.51-0.89)	0.57 (0.43-0.77)	0.001	0.32
Women	Median (% of total FA)	0.69	0.82	0.94	1.11		
	AO prevalence[4], n (%)	207 (41)	196 (39)	196 (39)	192 (38)		
	Crude OR[5] (95% CI)	1.00 (reference)	0.91 (0.71-1.17)	0.91 (0.71-1.17)	0.89 (0.69-1.14)	0.38	0.41
	Adjusted OR[5,6] (95% CI)[6]	1.00 (reference)	0.95 (0.74-1.23)	0.93 (0.72-1.20)	0.90 (0.69-1.16)	0.41	0.32
Eicosapentaenoic acid							
Men	Median (% of total FA)	1.16	1.65	2.13	3.08		
	AO prevalence[4], n (%)	143 (30)	143 (30)	148 (31)	115 (25)		
	Crude OR[5] (95% CI)	1.00 (reference)	1.00 (0.76-1.32)	1.05 (0.80-1.38)	0.74 (0.56-0.99)	0.0375	0.52
	Adjusted OR[5,6] (95% CI)[6]	1.00 (reference)	1.01 (0.76-1.35)	1.07 (0.81-1.43)	0.76 (0.57-1.03)	0.07	0.45
Women	Median (% of total FA)	1.24	1.66	2.13	3.07		
	AO prevalence[4], n (%)	179 (35)	218 (43)	192 (38)	202 (40)		
	Crude OR[5] (95% CI)	1.00 (reference)	1.38 (1.07-1.78)	1.12 (0.86-1.44)	1.22 (0.94-1.57)	0.44	0.45
	Adjusted OR[5,6] (95% CI)[6]	1.00 (reference)	1.46 (1.12-1.89)	1.30 (1.00-1.69)	1.46 (1.12-1.91)	0.0305	0.10
Docohexaenoic acid							
Men	Median (% of total FA)	0.63	0.81	0.95	1.19		
	AO prevalence[4], n (%)	147 (31)	138 (29)	149 (31)	115 (24)		
	Crude OR[5] (95% CI)	1.00 (reference)	0.91 (0.69-1.21)	1.02 (0.77-1.34)	0.71 (0.54-0.95)	0.0378	0.18
	Adjusted OR[5,6] (95% CI)[6]	1.00 (reference)	0.93 (0.70-1.24)	1.08 (0.81-1.43)	0.80 (0.59-1.08)	0.23	0.13
Women	Median (% of total FA)	0.67	0.83	0.97	1.19		
	AO prevalence[4], n (%)	227 (45)	207 (41)	194 (38)	163 (32)		
	Crude OR[5] (95% CI)	1.00 (reference)	0.85 (0.66-1.09)	0.76 (0.60-0.98)	0.59 (0.45-0.76)	<0.0001	0.96
	Adjusted OR[5,6] (95% CI)[6]	1.00 (reference)	0.94 (0.73-1.22)	0.89 (0.68-1.15)	0.73 (0.56-0.95)	0.0160	0.74

[1]FA, fatty acid; OR, odds ratio; [2]P for overall trend (P_{trend}) was evaluated using logistic regression models with sex-specific quartile median as exposure. [3]P for nonlinearity ($P_{non-linear}$) was evaluated using restricted cubic splines. [4]Abdominal obesity was defined as WC>88 cm in women and WC>102 cm in men. [5]OR and 95% CI were evaluated using logistic regression models. [6]Adjusted for physical activity, alcohol intake, education and smoking.
