# Supplementary material for: Serum Fatty Acids, Desaturase Activities and Abdominal Obesity – A Population-Based Study of 60-Year Old Men and Women
Source: PLoS One. 2017 Jan 26;12(1):e0170684. doi: 10.1371/journal.pone.0170684 (PMC5270324; doi:10.1371/journal.pone.0170684)
Supplement: S3 Table — (PDF) [file pone.0170684.s004.pdf]

S3 Table. Associations of serum fatty acids with anthropometric measures in men and women[1,2]

		Quartile of serum fatty acids					
		1	2	3	4	P_{trend}[3]	$P_{non-linear}$[4]
Palmitic acid (16:0, PA)							
SAD, cm	Men	21.1 (20.8-21.3)	21.2 (20.9-21.4)	21.7 (21.5-21.9)	22.0 (21.7-22.2)	<0.0001	0.0125
	Women	19.6 (19.4-19.9)	19.8 (19.5-20.0)	19.8 (19.6-20.0)	19.8 (19.6-20.1)	0.21	0.0493
WC, cm	Men	96.0 (95.1-96.9)	96.6 (95.7-97.5)	98.7 (97.8-99.6)	99.9 (99.0-100.9)	<0.0001	0.0118
	Women	85.5 (84.4-86.5)	86.3 (85.2-87.3)	86.8 (85.7-87.8)	87.1 (86.0-88.1)	0.0258	0.0168
WHR	Men	0.94 (0.94-0.95)	0.95 (0.94-0.95)	0.96 (0.95-0.96)	0.96 (0.96-0.97)	<0.0001	0.003
	Women	0.82 (0.82-0.83)	0.83 (0.82-0.83)	0.83 (0.82-0.84)	0.84 (0.83-0.84)	0.0005	0.12
Linoleic acid (18:2n6, LA)							
SAD, cm	Men	22.2 (21.9-22.4)	21.9 (21.7-22.1)	21.1 (20.9-21.4)	20.7 (20.5-20.9)	<0.0001	<0.0001
	Women	20.5 (20.2-20.7)	19.9 (19.7-20.1)	19.6 (19.3-19.8)	19.1 (18.8-19.3)	<0.0001	0.0018
WC, cm	Men	100.6 (99.6-101.5)	99.0 (98.1-99.9)	96.8 (95.8-97.7)	94.8 (93.9-95.8)	<0.0001	<0.0001
	Women	89.6 (88.6-90.6)	87.3 (86.3-88.3)	85.3 (84.3-86.3)	83.3 (82.3-84.3)	<0.0001	0.0003
WHR	Men	0.97 (0.96-0.97)	0.96 (0.95-0.96)	0.95 (0.94-0.95)	0.93 (0.93-0.94)	<0.0001	0.0004
	Women	0.85 (0.84-0.85)	0.83 (0.82-0.83)	0.82 (0.82-0.83)	0.81 (0.81-0.82)	<0.0001	0.12
α-linolenic acid							
SAD, cm	Men	21.7 (21.5-22.0)	21.5 (21.2-21.7)	21.5 (21.3-21.8)	21.2 (20.9-21.4)	0.0019	0.44
	Women	19.9 (19.7-20.2)	19.9 (19.6-20.1)	19.6 (19.3-19.8)	19.7 (19.5-19.9)	0.12	0.62
WC, cm	Men	99.1 (98.2-100.1)	97.9 (97.0-98.8)	97.7 (96.8-98.6)	96.5 (95.6-97.4)	0.0001	0.69
	Women	86.8 (85.8-87.8)	86.4 (85.4-87.4)	85.7 (84.7-86.7)	86.6 (85.6-87.6)	0.65	0.33
WHR	Men	0.96 (0.95-0.96)	0.95 (0.94-0.95)	0.95 (0.94-0.95)	0.95 (0.94-0.95)	0.0016	0.11
	Women	0.83 (0.82-0.83)	0.83 (0.82-0.83)	0.83 (0.82-0.83)	0.83 (0.83-0.84)	0.10	0.32
Eicosapentaenoic acid							
SAD, cm	Men	21.3 (21.0-21.5)	21.6 (21.4-21.9)	21.7 (21.4-21.9)	21.4 (21.1-21.6)	0.94	0.08
	Women	19.5 (19.3-19.8)	19.8 (19.6-20.0)	19.8 (19.6-20.0)	19.9 (19.7-20.2)	0.03	0.0305
WC, cm	Men	97.2 (96.2-98.1)	98.3 (97.4-99.2)	98.4 (97.5-99.4)	97.3 (96.4-98.3)	0.90	0.15
	Women	85.4 (84.4-86.4)	86.3 (85.3-87.3)	86.7 (85.6-87.7)	87.2 (86.2-88.2)	0.02	0.042
WHR	Men	0.95 (0.94-0.95)	0.96 (0.95-0.96)	0.95 (0.95-0.96)	0.95 (0.94-0.95)	0.31	0.90
	Women	0.83 (0.82-0.83)	0.83 (0.82-0.84)	0.83 (0.82-0.83)	0.83 (0.82-0.83)	0.78	0.56
Docohexaenoic acid							
SAD, cm	Men	21.4 (21.2-21.7)	21.5 (21.2-21.7)	21.6 (21.3-21.8)	21.4 (21.2-21.7)	0.97	0.10
	Women	19.9 (19.7-20.2)	19.8 (19.5-20.0)	19.7 (19.5-19.9)	19.6 (19.4-19.9)	0.08	0.85
WC, cm	Men	97.4 (96.4-98.3)	97.7 (96.8-98.7)	98.2 (97.3-99.1)	97.9 (97.0-98.8)	0.36	0.07
	Women	87.1 (86.0-88.1)	86.6 (85.6-87.6)	86.1 (85.1-87.1)	85.8 (84.8-86.8)	0.08	0.84
WHR	Men	0.95 (0.95-0.96)	0.95 (0.94-0.95)	0.95 (0.95-0.96)	0.95 (0.95-0.96)	0.89	0.17
	Women	0.83 (0.83-0.84)	0.83 (0.83-0.84)	0.82 (0.82-0.83)	0.82 (0.82-0.83)	0.0019	0.18

[1]FA, fatty acid; SAD, sagittal abdominal diameter; WC, waist circumference; WHR, waist-hip ratio. [2]Values are quartile means (95% CI) adjusted for physical activity, alcohol intake, education and smoking. [3]P for overall trend (P_{trend}) was evaluated using linear regression models with sex-specific quartile median as exposure. [4]P for nonlinearity ($P_{non-linear}$) was evaluated using restricted cubic splines.
