# Supplementary material for: Serum Fatty Acids, Desaturase Activities and Abdominal Obesity – A Population-Based Study of 60-Year Old Men and Women
Source: PLoS One. 2017 Jan 26;12(1):e0170684. doi: 10.1371/journal.pone.0170684 (PMC5270324; doi:10.1371/journal.pone.0170684)
Supplement: S4 Table — (PDF) [file pone.0170684.s005.pdf]

S4 Table. Associations of estimated desaturase activities with abdominal obesity in men and women[1]

	Quartile of estimated desaturase activity				P_{trend}[2]	$P_{non-linear}$[3]
	1	2	3	4		
SCD						
Men						
Median activity*	0.19	0.25	0.30	0.42		
AO prevalence, n (%)	74 (16)	107 (23)	174 (37)	194 (41)		
Crude OR (95% CI)	1.00 (reference)	1.58 (1.13-2.19)	3.13 (2.30-4.28)	3.75 (2.76-5.11)	<0.0001	<0.0001
Adjusted OR (95% CI)[6]	1.00 (reference)	1.54 (1.11-2.16)	3.08 (2.24-4.25)	3.65 (2.61-5.12)	<0.0001	<0.0001
Women						
Median activity*	0.23	0.29	0.36	0.47		
AO prevalence, n (%)	116 (23)	162 (32)	231 (46)	282 (56)		
Crude OR (95% CI)	1.00 (reference)	1.58 (1.20-2.09)	2.82 (2.15-3.70)	4.24 (3.23-5.57)	<0.0001	<0.0001
Adjusted OR (95% CI)[6]	1.00 (reference)	1.67 (1.25-2.22)	2.93 (2.22-3.87)	4.68 (3.51-6.25)	<0.0001	<0.0001
D5D						
Men						
Median activity*	6.77	8.16	9.48	11.47		
AO prevalence, n (%)	175 (37)	160 (34)	122 (26)	92 (19)		
Crude OR (95% CI)	1.00 (reference)	0.87 (0.67-1.14)	0.59 (0.49-0.78)	0.41 (0.31-0.55)	<0.0001	0.08
Adjusted OR (95% CI)[6]	1.00 (reference)	0.88 (0.67-1.16)	0.60 (0.45-0.80)	0.40 (0.30-0.55)	<0.0001	0.12
Women						
Median activity*	6.66	8.06	9.50	11.44		
AO prevalence, n (%)	256 (50)	225 (44)	167 (33)	143 (28)		
Crude OR (95% CI)	1.00 (reference)	0.78 (0.61-1.00)	0.48 (0.37-0.62)	0.39 (0.30-0.50)	<0.0001	0.0029
Adjusted OR (95% CI)[6]	1.00 (reference)	0.81 (0.63-1.04)	0.48 (0.37-0.62)	0.41 (0.31-0.53)	<0.0001	0.0017
D6D						
Men						
Median activity*	0.011	0.015	0.020	0.027		
AO prevalence, n (%)	100 (21)	119 (25)	136 (29)	194 (41)		
Crude OR (95% CI)	1.00 (reference)	1.25 (0.93-1.70)	1.50 (1.12-2.02)	2.60 (1.95-3.46)	<0.0001	0.33
Adjusted OR (95% CI)[6]	1.00 (reference)	1.21 (0.89-1.65)	1.44 (1.06-1.95)	2.39 (1.78-3.22)	<0.0001	0.33
Women						
Median activity*	0.011	0.016	0.021	0.028		
AO prevalence, n (%)	131 (26)	152 (30)	220 (43)	288 (57)		
Crude OR (95% CI)	1.00 (reference)	1.23 (0.93-1.62)	2.20 (1.69-2.87)	3.79 (2.91-4.94)	<0.0001	0.0022
Adjusted OR (95% CI)[6]	1.00 (reference)	1.18 (0.89-1.57)	2.09 (1.59-2.74)	3.55 (2.70-4.67)	<0.0001	0.0036

[1]D5D, Δ5-desaturase; D6D, Δ6-desaturase; OR, odds ratio; SCD, stearoyl-CoA desaturase; WC, waist circumference; WHR, waist-hip ratio. . [2]P for overall trend (P_{trend}) was evaluated using logistic regression models with sex-specific quartile median as exposure. [3]P for nonlinearity ($P_{non-linear}$) was evaluated using restricted cubic splines. [4]Abdominal obesity was defined as WC>88 cm in women and WC>102 cm in men. [5]OR and 95% CI were evaluated using logistic regression models. [6]Adjusted for physical activity, alcohol intake, education and smoking.
