# Supplementary material for: Serum Fatty Acids, Desaturase Activities and Abdominal Obesity – A Population-Based Study of 60-Year Old Men and Women
Source: PLoS One. 2017 Jan 26;12(1):e0170684. doi: 10.1371/journal.pone.0170684 (PMC5270324; doi:10.1371/journal.pone.0170684)
Supplement: S5 Table — (PDF) [file pone.0170684.s006.pdf]

S5 Table. Associations of estimated desaturase activities with anthropometric measures in men and women.[1,2]

		Quartile of serum fatty acids					
		1	2	3	4	P_{trend}[3]	$P_{non-linear}$[4]
SCD							
SAD, cm	Men	20.4 (20.2-20.7)	21.0 (20.8-21.3)	22.0 (21.7-22.2)	22.5 (22.2-22.7)	<0.0001	<0.0001
	Women	18.7 (18.5-18.9)	19.4 (19.1-19.6)	20.1 (19.9-20.4)	20.8 (20.6-21.1)	<0.0001	<0.0001
WC, cm	Men	93.8 (92.9-94.8)	96.5 (95.6-97.3)	100.0 (99.1-100.8)	101.0 (100.0-101.9)	<0.0001	<0.0001
	Women	81.4 (80.4-82.4)	85.0 (84.0-86.0)	88.1 (87.1-89.0)	91.1 (90.1-92.1)	<0.0001	<0.0001
WHR	Men	0.93 (0.93-0.94)	0.94 (0.94-0.95)	0.96 (0.96-0.97)	0.97 (0.96-0.97)	<0.0001	<0.0001
	Women	0.81 (0.80-0.81)	0.82 (0.82-0.83)	0.83 (0.83-0.84)	0.85 (0.85-0.86)	<0.0001	0.0015
D5D							
SAD, cm	Men	22.2 (22.0-22.4)	21.9 (21.7-22.1)	21.2 (21.0-21.4)	20.6 (20.4-20.8)	<0.0001	0.0005
	Women	20.6 (20.4-20.9)	19.9 (19.6-20.1)	19.4 (19.2-19.7)	19.1 (18.8-19.3)	<0.0001	0.0008
WC, cm	Men	100.3 (99.4-101.2)	99.2 (98.3-100.1)	96.8 (95.9-97.7)	95.0 (94.0-95.9)	<0.0001	0.0055
	Women	90.2 (89.2-91.2)	87.1 (86.1-88.0)	85.0 (84.1-86.0)	83.2 (82.2-84.2)	<0.0001	0.0002
WHR	Men	0.96 (0.96-0.97)	0.96 (0.95-0.96)	0.95 (0.94-0.95)	0.94 (0.93-0.94)	<0.0001	0.1024
	Women	0.85 (0.84-0.85)	0.83 (0.83-0.84)	0.82 (0.82-0.83)	0.81 (0.81-0.82)	<0.0001	0.0027
D6D							
SAD, cm	Men	20.7 (20.5-21.0)	21.2 (20.9-21.4)	21.7 (21.4-21.9)	22.3 (22.1-22.6)	<0.0001	0.0046
	Women	18.9 (18.7-19.2)	19.4 (19.1-19.6)	20.0 (19.8-20.2)	20.8 (20.5-21.0)	<0.0001	0.0061
WC, cm	Men	95.0 (94.1-95.9)	97.0 (96.1-97.9)	98.5 (97.6-99.4)	100.7 (99.8-101.7)	<0.0001	0.0044
	Women	82.7 (81.8-83.7)	84.5 (83.6-85.5)	87.7 (86.7-88.7)	90.6 (89.6-91.5)	<0.0001	0.0001
WHR	Men	0.94 (0.93-0.94)	0.95 (0.94-0.95)	0.96 (0.95-0.96)	0.96 (0.96-0.97)	<0.0001	0.0003
	Women	0.81 (0.80-0.82)	0.82 (0.81-0.82)	0.83 (0.83-0.84)	0.85 (0.84-0.86)	<0.0001	0.0004

[1]D5D, Δ5-desaturase; D6D, Δ6-desaturase; SAD, sagittal abdominal diameter; SCD, stearoyl-CoA desaturase; WC, waist circumference; WHR, waist-hip ratio.[2]Values are quartile means (95% CI) adjusted for physical activity, alcohol intake, education and smoking. [3]P for overall trend (P_{trend}) was evaluated using linear regression models with sex-specific quartile median as exposure. [4]P for nonlinearity ($P_{non-linear}$) was evaluated using restricted cubic splines.
